# Isolation and characterization of microsatellite markers and analysis of genetic variability in *Curculigo latifolia* Dryand

**DOI:** 10.1007/s11033-012-1853-z

**Published:** 2012-06-30

**Authors:** Nahid Babaei, Nur Ashikin Psyquay Abdullah, Ghizan Saleh, Thohirah Lee Abdullah

**Affiliations:** Department of Crop Science, Faculty of Agriculture, Universiti Putra Malaysia, 43400 Serdang, Selangor Malaysia

**Keywords:** SSR markers, 5′ and 3′ anchored ISSR, Genetic variation, Lemba, Polymorphism

## Abstract

Curculin, a sweet protein found in *Curculigo latifolia* fruit has great potential for the pharmaceutical industry. This protein interestingly has been found to have both sweet taste and taste-modifying capacities comparable with other natural sweeteners. According to our knowledge this is the first reported case on the isolation of microsatellite loci in this genus. Hence, the current development of microsatellite markers for *C. latifolia* will facilitate future population genetic studies and breeding programs for this valuable plant. In this study 11 microsatellite markers were developed using 3′ and 5′ ISSR markers. The primers were tested on 27 accessions from all states of Peninsular Malaysia. The number of alleles per locus ranged from three to seven, with allele size ranging from 141 to 306 bp. The observed and expected heterozygosity ranged between 0.00–0.65 and 0.38–0.79, respectively. The polymorphic information content ranged from 0.35 to 0.74 and the Shannon’s information index ranged from 0.82 to 1.57. These developed polymorphic microsatellites were used for constructing a dendrogram by unweighted pair group method with arithmetic mean cluster analysis using the Dice’s similarity coefficient. Accessions association according to their geographical origin was observed. Based on characteristics of isolated microsatellites for *C. latifolia* accessions all genotype can be distinguished using these 11 microsatellite markers. These polymorphic markers could also be applied to studies on uniformity determination and somaclonal variation of tissue culture plantlets, varieties identification, genetic diversity, analysis of phylogenetic relationship, genetic linkage maps and quantitative trait loci in *C. latifolia.*

**Electronic supplementary material:**

The online version of this article (doi:10.1007/s11033-012-1853-z) contains supplementary material, which is available to authorized users.

## Introduction

Lemba (*Curculigo latifolia* Dryand) a monocotyledonous perennial herb belongs to the Hypoxidaceae family. The genus *Curculigo* comprising of about 20 species is distributed in the tropical regions of Asia and Africa [1]. *C. latifolia* is widely spread in primary and secondary forests throughout Malaysia. Species confounded in Borneo are *C. racemose* and *C. orchioides*. Curculin, which is extracted from fruits of *C. latifolia* has been found to have a sweet taste with sweetness-modifying characteristics of natural sweeteners and has been shown to be a good low-calorie sweetener [2, 3]. It has been proven that curculin is up to 9,000 times sweeter than sucrose [4] and has antidiabetic properties [5]. Therefore, this plant has great potential for the pharmaceutical and food industries. Plants are currently being brought into cultivation. However, prior to cultivation, it is prudent to look at the fundamental knowledge of the population structure of this plant. The beneficial characteristics of the species can be further enhanced through plant breeding, but characterization of the available species in Malaysia is loose and considered necessary before any breeding work can commence. Determination of genetic diversity and population structure are prerequisites of breeding programs and a first step in the development and evaluation of plant genotypes.

Microsatellites or simple sequence repeats (SSRs) are tandemly repeated motifs of 1–6 nucleotides found in all prokaryotic and eukaryotic genomes [6]. Since microsatellites are co-dominants inherited, highly abundant, polymorphic, multiallelic, and reproducible with transferability characteristics, they become one of the most desirable markers for use in genetic studies [7]. Interestingly, SSR has been the marker of choice for assessment of genetic variability in many plant species such as commercial peach varieties [8], sugar beet [9], barley [10] and chickpea [11], analysis of phylogenetic relationship [12], marker assisted selection [13], construction of genetic linkage maps [14], and quantitative trait loci (QTL) [15]. Microsatellites may be identified by screening DNA databases, but for this genus no sequence information has been reported and no microsatellite markers were isolated and developed. It was therefore essential to develop microsatellites for *C. latifolia*. The knowledge generated could also be used in related species.

ISSR-PCR is an alternative strategy that has been devised to reduce the time invested in microsatellites isolation and to significantly increase yield [16] without the need for enrichment and/or hybridization screening [17]. Besides, the ISSR-PCR technique targets only those regions of the genome that are rich in microsatellite motifs [18, 19]. In plants, the construction of microsatellite markers with both 3′ and 5′ anchored ISSR-PCR strategy has been proven to be effective in producing polymorphic loci for different species such as, Canada thistle [20], wheat [21, 22], oil palm [23], Japanese persimmon [24], and turnip [25].

This study was performed with the objectives of to develop polymorphic microsatellite markers for *C. latifolia* using 5′ and 3′ anchored ISSR primers; and to determine the suitability of developed microsatellite markers for genetic variation study using constructing a dendrogram and demonstrating relationships among *C. latifolia* accessions.

## Materials and methods

### DNA extraction

Genomic DNA was extracted from the young leaf of 27 accessions from Peninsular Malaysia using GENE ^√^ ALL™ Plant SV Mini Kit (from General Biosystem, Seoul, Korea) following manufacturer’s instructions with the DNA concentration adjusted to 70 ng/μl.

### Microsatellite markers development

In this study an accession from Ringlet was used for PCR amplification using ISSR markers. Both 3′ and 5′ anchored ISSR primers were used for microsatellite markers development. The 3′ anchored ISSRs were UBC815 and UBC835 with sequences of (CT)_8_G and (AG)_8_YC, respectively. Three 5′ anchored ISSRs used were RAM1, BP8 and BP10 with sequences of YHY(CCA)_5,_ KKYHYHYHY(GTT)_5_ and KKDRDRD(TC)_10_ respectively, where Y = C/T, H = A/T/C, K = G/T, D = G/A/T and R = A/G.

PCR was carried out in a total volume of 25 μl including deionized water, 1 × PCR buffer plus MgSO_4_, 200 μM dNTP mix, 0.6 μM primer, 0.75 Pfu DNA polymerase and 70 ng/μl DNA. The thermal cycler with the touchdown thermal cycling protocol starting with three minutes of denaturing at 94°C was followed by the remaining thermal cycling protocol where temperature was set to 94°C for 40 s. The annealing step was started at 10°C above optimum annealing temperature for 30 s and then reduced by 1°C per cycle until optimum annealing temperature followed by 60 s extension time at 72°C. The program was followed by the remaining thermal cycling protocol where temperature was set to 95°C for 40 s, then at the primer’s optimum annealing temperature for 50 s, and extension at 72°C for 60 s, for a total of 30 cycles with a final ten minutes extension at 72°C. Amplified products were resolved via 2 % agarose gel, stained by ethidium bromide and visualized by UV-light.

Fragments ranging from 250 to 1,300 were purified using Gene JET™ PCR Purification Kit (Fermentas) and ligated into pCR^®^II-Blunt-TOPO^®^ vector (Zero Blunt^®^ TOPO^®^ PCR Cloning Kit, Invitrogen^®^) following manufacturer’s instructions and then transformed into *Escherichia coli* DH5α component cells. Transformed clones were grown overnight in selective media (LB-Amp). Ten randomly selected recombinant clones were grown overnight in LB broth and plasmids DNA were extracted using PureLink™ Quick Plasmid Miniprep Kit (Invitrogen^®^). Extracted plasmid DNA of recombinant clones was sequenced. Microsatellite motifs were screened using Microsatellite Repeat Finder—Online Bioinformatic Tools and primers were designed using PRIMER3 Version 0.4.0 (http://frodo.wi.mit.edu/primer3/). The major parameters for primer design were set as follows: primer length from 20 to 25 nucleotides, PCR products size from 140 to 310 bp, annealing temperatures at 55.5–61.5°C and GC content of between 40 and 60 %.

### Microsatellites genotyping

**Fig. 1:**
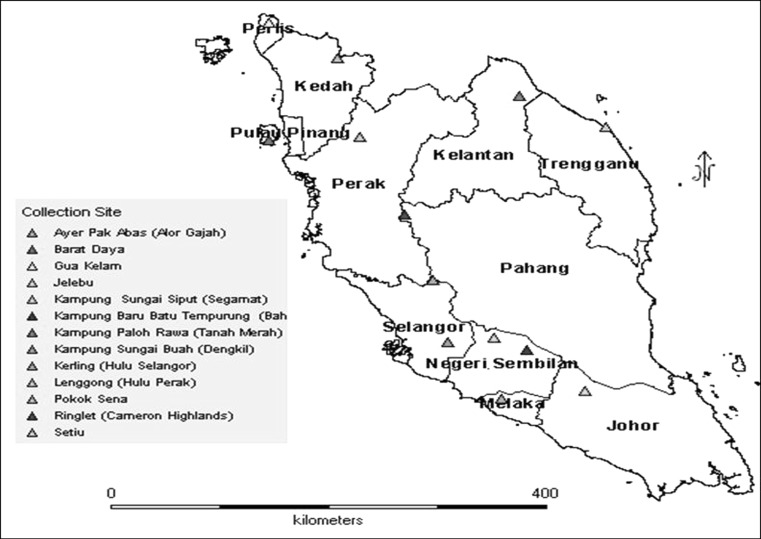
DIVA-GIS mapping of collection sites of *C. latifolia* from Peninsular Malaysia

The developed primers were tested on 27 accessions of *C. latifolia*, 16 of which were collected from Jelebu, Negeri Sembilan (accession 13–27) and the rest from all other states in Peninsular Malaysia. The locations of *C. latifolia* accessions used for genetic variability study are illustrated using DIVA-GIS software version 7.2.1 (Fig. 1). PCR amplifications were performed in 25 μl volumes containing 0.75 U Pfu DNA polymerase (Fermentas), 1 × PCR buffer plus MgSO_4_, 200 μM dNTP mix, 0.4 μM of each primer set, and 70 ng template DNA. The PCR program used was as mention previously. Amplification products were resolved via 4 % methaphor agarose gel using ethidium bromide staining. A 50-bp DNA ladder (Fermentas) was used as a reference marker to determine the size of alleles.

### Data analysis

The observed (N_a_) and effective (N_e_) number of alleles, observed (H_o_) and expected (H_E_) heterozygosity, Shannon’s information index (I) and Hardy–Weinberg equilibrium (HWE) were calculated with the software POPGENE Version 1.31 [26]. Polymorphism information content (PIC), a measure of allelic diversity at a locus was calculated using the following formula.

$$ PIC = 1 - \sum\limits_{i = 1}^{n} {p_{i}^{2} } - \sum\limits_{i}^{n - 1} {\sum\limits_{j = i + 1}^{n} {2p_{i}^{2} p_{j}^{2} } } $$

where *p*_*i*_ is the frequency of the *i*th allele, and *n* is the number of alleles [27].

The binary data attained from scoring of microsatellite markers were analyzed using NTSYS-pc 2.1 in order to reveal the genetic variability and associations among *C. latifolia* accessions. The coefficients of genetic similarity were computed using Dice’s similarity coefficient. This similarity matrix was used to create a dendrogram using the unweighted paired group method using arithmetic average (UPGMA). Principal component analysis (PCA) was also carried out to explore associations among accessions [28].

Gap statistics was computed to estimate the number of clusters in the dendrogram [29]. The gap statistics were analyzed using R software version 2.15.0 using matrix data.

## Results

### Sequence analysis of cloned fragments

Among the 68 randomly-selected clones of inserted fragments amplified from the five ISSR markers, a total of 50 clones were selected for sequence analyses. The average size of the inserts obtained from the sequencing was 532 bp. All 50 recombinant clones which were sequenced contained microsatellite motifs at both ends and the presence of additional internal microsatellites observed in some clones implied clustering of microsatellites in several genomic regions. Among these, 36 clones (72 %) had unique sequences that were different from each other. The remaining 14 clones displayed the same sequences present in the selected unique clones. Since fragments with different sizes were selected for sequencing, this resulted in the isolation of mostly (72 %) unique microsatellite loci and a few redundant sequences. Nucleotide sequence data with the microsatellite loci are available in the GenBank databases under the accessions number reported in Table 1. With the use of ISSR primers for selective amplification of microsatellite-rich DNA fragments, all clones contained reverse complementary repeated motifs at the two ends. The clones containing CT, AG, CCA, GTT and TC motifs at the 5′ ends carried GA, TC, GGT, CAA and AG motifs respectively, at the 3′ ends.

**Table 1:** Characterization of the microsatellite loci in 27 *C. latifolia* accessions

Locus	Gene bank accession no.	Repeat motif	Primer sequence (5′–3′)	Ta (°C)	N_a_/N_e_	Allele size range (bp)	Ho	H_E_	PIC	I
Curla1	JN976991	(CA)4…(A)16G(A)6	F:CCA ACT ATC CTT TCC CGA CA	59.7	7/4.4	218–288	0.18	0.79	0.74	0.94
			R:TGG GTA GGG GTC CTC TCT CT							
Curla5	JN976994	(CAA)5…(AT)3	F:CTC TCT CTC TGT GCC CCA AG	55.5	6/4.4	171–189	0.23	0.79	0.74	0.82
			R:CGC ACC ATA CGT TTG TTT GA							
Curla10	JN976998	(TTAGA)3…(TC)3	F:GAG AGC CAC GAG TAA AGA GTC A	57.4	4/1.6	141–157	0.00	0.38	0.35	1.20
			R:AAG GCT TAC ACT AAT GAT TTG CTT							
Curla12	JN976999	(TCT)3…(CAA)3…(TCT)5	F:CCG GTT GAG GAT ACA AAT GG	57.4	7/4.5	147–185	0.33	0.79	0.74	1.57
			R:GGA CCA GCT GAG CAT TGA TT							
Curla13	JN976999	(TCT)3…(CAA)3…(TCT)5…(CT)4…(AT)3	F:CCG GTT GAG GAT ACA AAT GG	55.5	7/4.4	237–278	0.37	0.78	0.74	1.23
			R:AAG CGG GAG AGG CAT TTA TT							
Curla14	JN977000	(TC)3…(A)8	F:GAG AGA GAG AGA GCC CAGCA	57.4	4/2.0	191–208	0.00	0.52	0.47	1.31
			R:TTG GCC ATG AAA TTT TGT CC							
Curla28	JN977012	(GAA)7	F:ACCCGAATTCCTCTCCAACT	58.4	4/2.5	251–277	0.00	0.61	0.54	0.89
			R:GGCTCTCTCTTGCTCCCATA							
Curla29	JN977016	(TC)3…(GGA)3…(CTC)4…(TCA)3	F:TCCAGCATAGGGATCTCCAC	59.4	3/1.8	295–306	0.00	0.46	0.39	0.92
			R:GGTTAGCCCCTGACAAGACA							
Curla30	JN977018	(TA)10…(TA)3	F:AATGGCGATTTCTTGGTGAG	55.5	7/4.1	166–226	0.65	0.77	0.72	1.51
			R:TGGAGAGATGGGGATAGCTG							
Curla33	JN977022	(GTT)3	F:CCTAGTGGGCGAAGATACCA	59.4	4/2.5	189–215	0.07	0.61	0.55	0.91
			R:GATCACTCGTTGGGGACAGT							
Curla34	JN977024	(AG)3…(CT)3…(GT)3	F:CACCGATGGTCTAGGAGGAG	61.5	3/2.1	219–227	0.00	0.55	0.47	1.07
			R:CTCTCGAGCACACTGACAGG							

*Ta* annealing temperature (°C), *Na* number of observed alleles, *Ne* effective number of alleles, *Ho* observed heterozygosity, *He* expected heterozygosity, *PIC* polymorphic information content, *I* Shannon’s Information index]

A total of 235 microsatellite regions were identified from the sequencing results consisting of different microsatellite core units. Among all microsatellite motifs found, the dinucleotide core was the most frequent with 121 motifs, followed by mononucleotides and trinucleotides with 64 and 47 motifs, respectively. In contrast, only two and one tetranucleotide and pentanucleotide motifs respectively were found, and no hexanucleotide microsatellite motif was found among all sequences. Among the selected sequenced clones, 86.1 % (31 out of 36 unique sequences) contained internally located microsatellite motifs in addition to those at the ends with variable flanking regions on both sides of the motifs. Based on Weber’s (1990) classification rules [30], among the 36 unique sequences, 99 perfect microsatellites without interruption, five compound repeat sequences with adjacent tandem microsatellites of a different sequence, and 10 imperfect/interrupted compound microsatellites with one or more interruptions in the run of repeats were found centrally located at the sequences.

### Microsatellite polymorphism within *C. latifolia* accessions

**Fig. 2:**
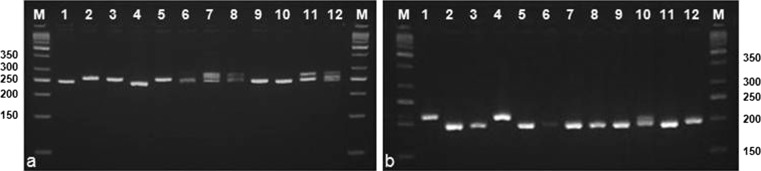
The genetic profiles of 12 accessions of *C. latifolia* using **a** primer Curla13 and **b** Curla 33 (M: 50 bp DNA ladder (fermentas); 1–12: Accessions of *C. latifolia*)

Of the total 34 designed primer pairs on the flanking regions of both perfect and interrupted compound microsatellites, 22 loci successfully amplified *C. latifolia* genomic DNA and out of these 22 primer pairs, 11 primer sets were polymorphic (Table 1). A total of 56 alleles were found at these 11 polymorphic loci and the total effective number of alleles was 34.3. The number of observed alleles (Na) per locus ranged from three (Curla29 and Curla34) to seven (Curla1, Curla12, Curla13, and Curla30) with an average of 5.1. The effective number of alleles ranged from 1.6 (Curla10) to 4.5 (Curla12) with an average of 3.12. The allele size was between 141 and 306 bp which conformed to the expected allele size parameter for microsatellite markers development. The genetic profiles of some accessions are illustrated in Fig. 2. The observed heterozygosity ranged from 0.00 (Curla10, Curla14, Curla28, Curla29, and Curla34) to 0.65 (Curla30), whereas the expected heterozygosity ranged from 0.38 (Curla10) to 0.79 (Curla1, Curla5, and Curla12). The polymorphic information content (PIC) value was between 0.35 (Curla10) and 0.74 (Curla1, Curla5, Curla12, and Curla13), with an average of 0.59. Thus, four of the loci (Curla10, Curla14, Curla29, and Curla34) were moderately polymorphic (0.25 < PIC < 0.5) and the eight remaining loci were highly polymorphic (0.5 < PIC), while none of the loci showed low polymorphism. The mean Shannon’s Information index (I) was 1.13, and ranged from 0.82 (Curla5) to 1.57 (Curla12). All loci deviated significantly from Hardy–Weinberg equilibrium.

### Genotyping and variability analysis

**Fig. 3:**
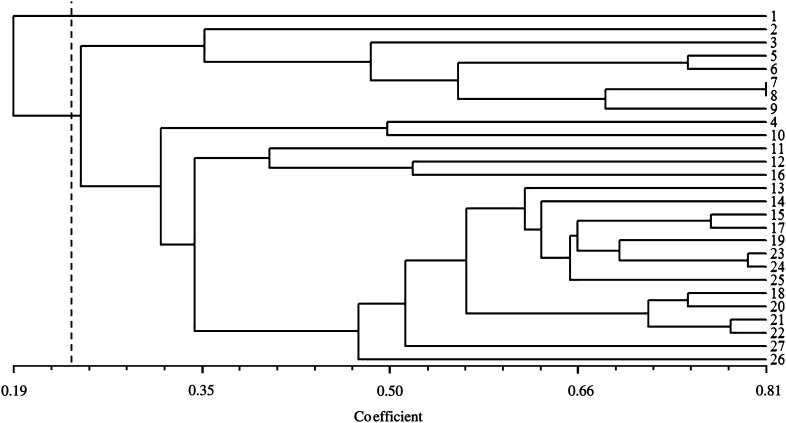
Dendrogram based on Dice’s similarity coefficient indicating relationships among accessions of *C. latifolia*

**Fig. 4:**
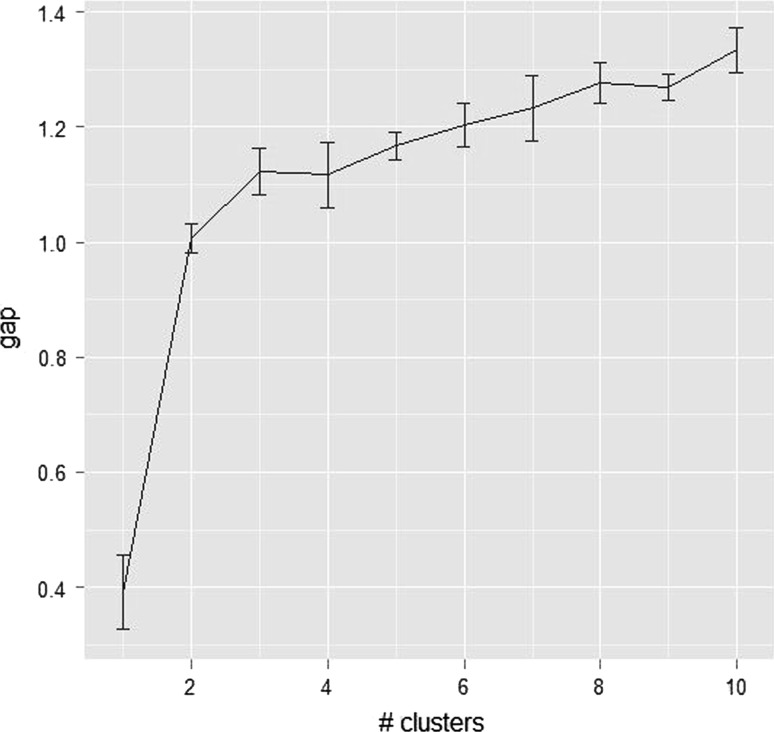
The gap curve indicated number of clusters revealed

The allelic data obtained with the 11 primer pairs across 27 accessions was scored and computed to obtain the Dice’s similarity coefficient. The relationships among these accessions using Dice’s similarity coefficients are indicated in Additional file 1. The average value of Dice’s similarity coefficient calculated was 0.44. The highest similarity coefficient, 0.81, was between accessions 7 and 8 from Pokok Sena and Gua Kelam and the lowest, 0.00, was between accessions 1 and 5 from Kerling and Lenggong respectively. Based on Dice’s similarity coefficient, a dendrogram was generated using UPGMA cluster analysis (Fig. 3). The similarity matrix also was used to estimate number of clusters using gap statistics analysis. It was found from results that a gap was observed between K = 1 and K = 2 (Table 2). This indicated that the dendrogram clearly distinguishes accessions surveyed into two clusters based on the calculated gap statistics (Fig. 4). The accession from Kerling, presented the lowest similarity values compared to those from the main group and it was placed as a cluster individually. The dendrogram revealed that accessions from neighboring states or geographically close locations almost grouped together indicating their genetic similarity. All accessions from Jelebu except accession 16 clustered into one sub-group on the similarity coefficient of 0.47. This indicated that accessions collected from one location was almost similar than accessions taken from different states.

**Table 2:** Gaps calculated for estimating number of clusters in the dendrogram

K	1	2	3	4	5	6	7	8	9	10
Gap	0.403	1.003	1.119	1.151	1.171	1.191	1.218	1.257	1.294	1.327

*K* number of cluster

**Fig. 5:**
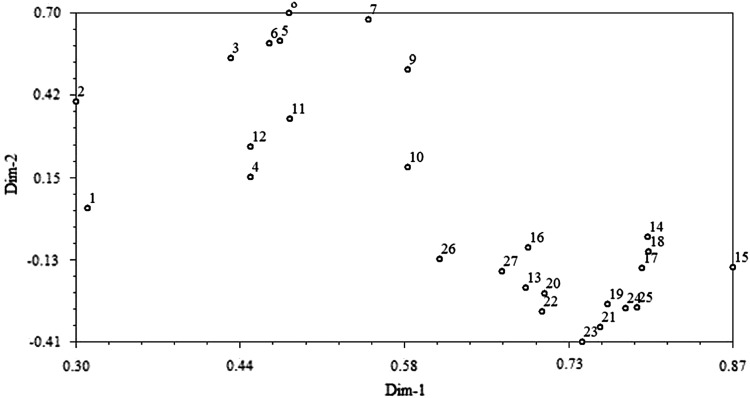
Two-dimensional plot of principle components of SSR marker data among *C. latifolia* accessions (Dim-1, Dim-2: the two axes represent the first two principle components; *labels* in the graph show the collection site of *C. latifolia*)

The binary data generated from the 27 *C. latifolia* accessions were also subjected to principal component analysis (PCA) to visualize accessions in a multivariate space. The first two principal components (PCs) extracted a cumulative of 53.80 % of the variance in the *C. latifolia* accessions. The first two components contributed 40.80 and 13.00 % of the total variation, respectively. The distribution of the accessions in the two-dimensional graph derived based on the first two principle components shows precisely the genetic status and association of accessions (Fig. 5). Results from both clustering and principle component analysis grouped *C. latifolia* accessions from Jelebu into one group which corresponded well to the close location (Fig. 3).

## Discussion

As indicated earlier the genus *Curculigo*, although widely distributed in Malaysia, Indonesia and Brunei, has not been characterized for genomic sequences and microsatellite development. Therefore, these developed microsatellite markers for *C. latifolia* could facilitate future population genetic studies and breeding programs for this plant and related species.

Microsatellite markers are characterized by a high degree of variability making them powerful tools for population genetic analyses [11]. The conventional protocols used for the isolation of microsatellites are cost, time and labor intensive and the efficiency of microsatellite isolation is low [16]. The primer extension strategy has been proven to be useful for the isolation of dinucleotide repeat microsatellites. Although enrichment method is more desirable than traditional, it is still time consuming because of many several steps and needs more investigation to gain tri- or tetra nucleotide containing microsatellites [31]. The isolation of microsatellites from plants is technically more demanding as their frequency in plants is relatively low comparing to animal genomes [32]. To overcome these limitations ISSR-PCR technique as targets only those regions of the genome those are rich in microsatellite motifs is a desirable strategy. This technique was highly successful, as over 86 % of unique clones obtained, contained internally located microsatellite motifs in addition to those at the ends.

In this study using ISSR-PCR technique, 11 polymorphic microsatellites have been developed and screened in 27 accessions of *C. latifola*. All the variability parameters calculated for the microsatellites described in the study indicated that microsatellites will become a useful tool for genetic variation studies, genotype identification and similarity analysis in *C. latifolia.* The average of 5.1 observed alleles and 3.12 of effective alleles per locus was detected in this study. The difference between average number of observed alleles and effective number of alleles was due to the uneven frequency of each allele [33]. PIC provides an estimate of discriminatory power of a marker to differentiate genotypes based on both the number of alleles expressed and their relative frequencies [34]. The average of PIC value was 0.59 which indicate an isolation of highly polymorphic microsatellites. This was consistent with findings on potential applications of ISSR-PCR technique in developing high polymorphic microsatellites in *Sphagnum capillifolium* with low level of genetic variation [17]. Overall genetic variability for the accessions studied, represented by Shannon’s indexes, was particularly high with the average of 1.13. The high value of Shannon’s information index represents the effectiveness of microsatellite loci to reveal the variation. The results indicated that all loci deviated significantly from HWE. Possible explanations for deviations of loci from HWE are heterozygote deficiency in loci [33], population size, and propagation through rhizomes.

The values obtained with Dice’s coefficient indicated that the extent of genetic variability among accessions varies, but that in most cases, genetic similarity is higher among accessions from one location or neighboring states. The highest Dice’s similarity coefficient (0.81) was found between accessions 7 and 8, indicating that they had almost the same genetic constituents based on the 11 microsatellite primers used. The lowest similarity coefficient (0.00) was found between accessions 1 and 5 which indicates that they were relatively remote in relationship.

The high level of genetic polymorphism was clearly evident from the dendrogram. *C. latifolia* is reported to be a cross-pollinating species [1]. The relatively high level of polymorphism could be due to cross pollination in this species, however low level of variation among accessions from confined population of a small size could be referred to propagation through rhizome in the species. The low genetic diversity among accessions of *C. latifolia* taken from one location also reported as a result of vegetative propagation through rhizome [35]. The rhizome propagation theoretically has a similar effect in population genetic structure as strict selfing [36].

The two-dimensional graph of accessions differentiation was revealed by PCA. This type of graphical illustration enables the assessment of the population structure and geometric distances among all of the accessions in the study [37]. The distribution of the accessions in the two-dimensional graph based on the first two principle components was similar to that obtained from cluster analysis, where all accessions collected from one location in Jelebu were distinctly separated from other accessions.

## Conclusion

In conclusion, 11 polymorphic microsatellite markers in *C. latifolia* were developed by the 5′ and 3′ anchored PCR technique. All the loci showed considerable variation in the population of this plant collected from within Malaysia. These results indicated that microsatellite primers tested could clearly distinguish the different sets of genotypes. The characteristics of these loci provide useful information for further studies on population genetics, assessment of genetic stability and somaclonal variation, construction of genetic linkage maps and mapping of economically quantitative trait loci, estimation of genetic diversity and divergence in *C.* *latifolia* and related plants. The use of these microsatellite markers will also facilitate the management and exploration of genetic resources of Hypoxidaceae in the lower Asparagales and assist in their genetic improvement to some extent.

## Electronic supplementary material

Below is the link to the electronic supplementary material.

(DOCX 23 kb)
