# Supplementary material for: Isolation and characterization of microsatellite markers and analysis of genetic variability in Curculigo latifolia Dryand
Source: Mol Biol Rep. 2012 Jun 30;39(11):9869–77. doi: 10.1007/s11033-012-1853-z (PMC3459080; doi:10.1007/s11033-012-1853-z)
Supplement: Supplementary file 1 — (DOCX 23 kb) [file 11033_2012_1853_MOESM1_ESM.docx]

**Isolation and characterization of microsatellite markers and analysis of genetic variability in *Curculigo latifolia* Dryand**

**Nahid Babaei, Nur Ashikin Psyquay Abdullah, Ghizan bin Saleh, Thohirah Lee Abdullah**

Department of Crop Science, Faculty of Agriculture, Universiti Putra Malaysia, 43400 UPM Serdang, Selangor, Malaysia.

Email address: NB; [nahid.babaei@yahoo.com](mailto:nahid.babaei@yahoo.com);

|  | 1 | 2 | 3 | 4 | 5 | 6 | 7 | 8 | 9 | 10 | 11 | 12 | 13 | 14 | 15 | 16 | 17 | 18 | 19 | 20 | 21 | 22 | 23 | 24 | 25 | 26 | 27 |
| --- | --- | --- | --- | --- | --- | --- | --- | --- | --- | --- | --- | --- | --- | --- | --- | --- | --- | --- | --- | --- | --- | --- | --- | --- | --- | --- | --- |
| 1 | 1.00 |  |  |  |  |  |  |  |  |  |  |  |  |  |  |  |  |  |  |  |  |  |  |  |  |  |  |
| 2 | 0.19 | 1.00 |  |  |  |  |  |  |  |  |  |  |  |  |  |  |  |  |  |  |  |  |  |  |  |  |  |
| 3 | 0.09 | 0.38 | 1.00 |  |  |  |  |  |  |  |  |  |  |  |  |  |  |  |  |  |  |  |  |  |  |  |  |
| 4 | 0.45 | 0.19 | 0.35 | 1.00 |  |  |  |  |  |  |  |  |  |  |  |  |  |  |  |  |  |  |  |  |  |  |  |
| 5 | 0.00 | 0.42 | 0.56 | 0.22 | 1.00 |  |  |  |  |  |  |  |  |  |  |  |  |  |  |  |  |  |  |  |  |  |  |
| 6 | 0.10 | 0.32 | 0.38 | 0.10 | 0.75 | 1.00 |  |  |  |  |  |  |  |  |  |  |  |  |  |  |  |  |  |  |  |  |  |
| 7 | 0.16 | 0.43 | 0.62 | 0.24 | 0.60 | 0.61 | 1.00 |  |  |  |  |  |  |  |  |  |  |  |  |  |  |  |  |  |  |  |  |
| 8 | 0.17 | 0.35 | 0.48 | 0.33 | 0.60 | 0.55 | 0.81 | 1.00 |  |  |  |  |  |  |  |  |  |  |  |  |  |  |  |  |  |  |  |
| 9 | 0.20 | 0.19 | 0.40 | 0.30 | 0.44 | 0.56 | 0.64 | 0.73 | 1.00 |  |  |  |  |  |  |  |  |  |  |  |  |  |  |  |  |  |  |
| 10 | 0.08 | 0.17 | 0.40 | 0.50 | 0.40 | 0.48 | 0.30 | 0.31 | 0.36 | 1.00 |  |  |  |  |  |  |  |  |  |  |  |  |  |  |  |  |  |
| 11 | 0.24 | 0.17 | 0.38 | 0.16 | 0.30 | 0.35 | 0.43 | 0.37 | 0.55 | 0.22 | 1.00 |  |  |  |  |  |  |  |  |  |  |  |  |  |  |  |  |
| 12 | 0.29 | 0.18 | 0.19 | 0.29 | 0.11 | 0.21 | 0.43 | 0.35 | 0.38 | 0.35 | 0.43 | 1.00 |  |  |  |  |  |  |  |  |  |  |  |  |  |  |  |
| 13 | 0.33 | 0.09 | 0.24 | 0.25 | 0.21 | 0.19 | 0.22 | 0.15 | 0.29 | 0.23 | 0.30 | 0.18 | 1.00 |  |  |  |  |  |  |  |  |  |  |  |  |  |  |
| 14 | 0.17 | 0.18 | 0.40 | 0.42 | 0.42 | 0.27 | 0.37 | 0.31 | 0.38 | 0.38 | 0.37 | 0.27 | 0.62 | 1.00 |  |  |  |  |  |  |  |  |  |  |  |  |  |
| 15 | 0.09 | 0.10 | 0.25 | 0.26 | 0.33 | 0.38 | 0.38 | 0.32 | 0.50 | 0.56 | 0.31 | 0.29 | 0.64 | 0.72 | 1.00 |  |  |  |  |  |  |  |  |  |  |  |  |
| 16 | 0.25 | 0.00 | 0.24 | 0.33 | 0.20 | 0.18 | 0.30 | 0.31 | 0.36 | 0.31 | 0.37 | 0.52 | 0.54 | 0.62 | 0.56 | 1.00 |  |  |  |  |  |  |  |  |  |  |  |
| 17 | 0.16 | 0.00 | 0.23 | 0.24 | 0.20 | 0.26 | 0.36 | 0.30 | 0.45 | 0.44 | 0.36 | 0.43 | 0.59 | 0.59 | 0.77 | 0.74 | 1.00 |  |  |  |  |  |  |  |  |  |  |
| 18 | 0.14 | 0.15 | 0.28 | 0.21 | 0.26 | 0.32 | 0.39 | 0.33 | 0.40 | 0.53 | 0.39 | 0.38 | 0.47 | 0.60 | 0.76 | 0.47 | 0.65 | 1.00 |  |  |  |  |  |  |  |  |  |
| 19 | 0.26 | 0.19 | 0.33 | 0.43 | 0.22 | 0.10 | 0.23 | 0.16 | 0.20 | 0.40 | 0.31 | 0.19 | 0.56 | 0.64 | 0.58 | 0.56 | 0.62 | 0.55 | 1.00 |  |  |  |  |  |  |  |  |
| 20 | 0.23 | 0.24 | 0.07 | 0.23 | 0.18 | 0.26 | 0.21 | 0.21 | 0.33 | 0.36 | 0.21 | 0.16 | 0.43 | 0.50 | 0.59 | 0.36 | 0.48 | 0.75 | 0.52 | 1.00 |  |  |  |  |  |  |  |
| 21 | 0.24 | 0.09 | 0.15 | 0.24 | 0.10 | 0.18 | 0.21 | 0.15 | 0.27 | 0.44 | 0.21 | 0.17 | 0.44 | 0.52 | 0.69 | 0.44 | 0.64 | 0.77 | 0.69 | 0.76 | 1.00 |  |  |  |  |  |  |
| 22 | 0.24 | 0.17 | 0.15 | 0.24 | 0.10 | 0.17 | 0.21 | 0.15 | 0.27 | 0.37 | 0.21 | 0.17 | 0.37 | 0.52 | 0.62 | 0.44 | 0.57 | 0.65 | 0.62 | 0.69 | 0.79 | 1.00 |  |  |  |  |  |
| 23 | 0.08 | 0.09 | 0.16 | 0.25 | 0.21 | 0.10 | 0.15 | 0.08 | 0.19 | 0.38 | 0.15 | 0.18 | 0.62 | 0.54 | 0.72 | 0.54 | 0.67 | 0.53 | 0.72 | 0.43 | 0.67 | 0.59 | 1.00 |  |  |  |  |
| 24 | 0.09 | 0.19 | 0.17 | 0.26 | 0.33 | 0.19 | 0.23 | 0.16 | 0.30 | 0.32 | 0.23 | 0.19 | 0.64 | 0.64 | 0.75 | 0.48 | 0.62 | 0.55 | 0.67 | 0.59 | 0.54 | 0.46 | 0.80 | 1.00 |  |  |  |
| 25 | 0.26 | 0.10 | 0.25 | 0.35 | 0.22 | 0.19 | 0.23 | 0.16 | 0.30 | 0.40 | 0.31 | 0.19 | 0.64 | 0.64 | 0.75 | 0.48 | 0.54 | 0.62 | 0.67 | 0.59 | 0.69 | 0.62 | 0.64 | 0.67 | 1.00 |  |  |
| 26 | 0.24 | 0.35 | 0.08 | 0.16 | 0.30 | 0.26 | 0.29 | 0.15 | 0.27 | 0.15 | 0.29 | 0.26 | 0.44 | 0.52 | 0.46 | 0.30 | 0.36 | 0.39 | 0.38 | 0.48 | 0.43 | 0.57 | 0.52 | 0.54 | 0.62 | 1.00 |  |
| 27 | 0.18 | 0.19 | 0.00 | 0.18 | 0.22 | 0.30 | 0.32 | 0.25 | 0.30 | 0.25 | 0.16 | 0.38 | 0.50 | 0.50 | 0.61 | 0.50 | 0.48 | 0.43 | 0.52 | 0.54 | 0.40 | 0.32 | 0.58 | 0.78 | 0.52 | 0.48 | 1.00 |

**Additional file 1.** Relationships among 27 *Cueculigo latifolia* accessions using Dice’s similarity coefficients
